# Season-adjusted longitudinal changes in serum 25-hydroxyvitamin D during pegylated interferon add-on to ongoing tenofovir alafenamide therapy in chronic hepatitis B: a real-world cohort study

**DOI:** 10.3389/fmed.2026.1976727

**Published:** 2026-09-30

**Authors:** Ning Han, Huan Liu, Xia Zhu

**Affiliations:** 1 Center of Infectious Diseases, West China Hospital of Sichuan University, Chengdu, China; 2 Laboratory of Infectious and Liver Disease, Institute of Infectious Diseases, West China Hospital, Sichuan University, Chengdu, China; 3 Division of Infectious Diseases, State Key Laboratory of Biotherapy, Sichuan University, Chengdu, China

**Keywords:** 25-hydroxyvitamin D, chronic hepatitis B, pegylated interferon, real-world cohort study, tenofovir alafenamide

## Abstract

**Objectives:**

To characterize serum 25-hydroxyvitamin D [25(OH)D] trajectories during peginterferon alfa-2b (Peg-IFN) add-on to ongoing tenofovir alafenamide (TAF) therapy in chronic hepatitis B and explore associations with hepatitis B surface antigen (HBsAg).

**Methods:**

This retrospective cohort identified 203 consecutive patients, of whom 190 contributed longitudinal data through week 48. The primary random-intercept mixed-effects model used categorical visit time and adjusted for seasonality, age, and sex. Continuous-time, random-slope, visit-timing, and HBsAg-censoring analyses assessed sensitivity.

**Results:**

The primary adjusted week-48 25(OH)D difference was −7.9 nmol/L (95% CI, −9.8 to −6.0) (*P* < 0.001); the secondary continuous-time estimate was −8.3 nmol/L over 48 weeks. Observed concentrations declined through week 36 and partially rebounded at week 48. The proportion below 47.7 nmol/L increased from 21.1% to 34.7%. The adjusted total serum calcium trend was −0.061 mmol/L (95% CI, −0.074 to −0.049). Triglycerides increased while cholesterol fractions decreased. At week 48, 104/190 patients (54.7%) had an HBsAg decline of at least 1 log10 and 50/190 (26.3%) had visit-specific HBsAg loss. The concurrent 48-week change correlation was weak (*ρ* = 0.150; FDR-adjusted *P* = 0.117), and the magnitude of 25(OH)D decline was not independently associated with either binary endpoint. Within patients, each 10 nmol/L increase in 25(OH)D was associated with a 0.068-log10 IU/mL increase in concurrent HBsAg (95% CI, 0.020 to 0.116; *P* = 0.006).

**Conclusions:**

Serum 25(OH)D showed a non-linear decline during 48 weeks of follow-up, and the week-48 difference remained evident after adjustment for seasonality. However, without a concurrent comparator, the observed change cannot be attributed specifically to Peg-IFN, TAF, or their combination. Exploratory analyses suggested only a weak concurrent relationship between 25(OH)D and HBsAg, without evidence of prediction or causality. The clinical relevance of the observed changes in 25(OH)D and total serum calcium warrants further prospective investigation.

## Introduction

Chronic hepatitis B virus (HBV) infection remains a major cause of cirrhosis, hepatocellular carcinoma, and liver-related mortality. Current treatment for chronic hepatitis B (CHB) relies primarily on potent nucleos(t)ide analogues, including tenofovir alafenamide (TAF). In selected patients, pegylated interferon (Peg-IFN) may be administered in combination with or sequentially after antiviral therapy to enhance immune-mediated viral control and increase the likelihood of hepatitis B surface antigen (HBsAg) decline or loss (1, 2). Although sustained virological suppression can be achieved in most patients, functional cure remains uncommon. Because CHB generally requires long-term management, treatment assessment should extend beyond virological endpoints to changes that may affect patients' overall health during therapy, including nutritional status, metabolic health, and calcium homeostasis.

Vitamin D status is commonly assessed by measuring circulating 25-hydroxyvitamin D [25(OH)D] concentrations (3). In addition to its established roles in calcium and phosphate homeostasis and bone metabolism, vitamin D has been implicated in immune responses to infection. Its effects in human viral infections, however, appear to be complex, and no consistent causal relationship has been established (4). Studies in patients with CHB have examined associations between vitamin D status, HBV replication, and HBV-related liver disease (5–9). In a baseline biomarker analysis within a randomized CHB treatment trial, Chan et al. found that associations between pretreatment vitamin D and week-48 virological outcomes were no longer significant after covariate adjustment, although an association with normal ALT remained, leaving uncertain whether vitamin D is mechanistically involved in treatment response or instead reflects underlying host or disease characteristics (6). Interpretation of total circulating 25(OH)D in liver disease is further complicated by hepatic hydroxylation and its binding to vitamin D-binding protein and albumin; hepatic dysfunction and inflammatory changes may therefore influence measured total 25(OH)D concentrations (3). Much of the other evidence concerns cross-sectional or baseline measurements; it does not establish how 25(OH)D changes longitudinally after Peg-IFN is added to ongoing TAF therapy.

TAF provides durable virological suppression and has a more favorable renal and bone safety profile than tenofovir disoproxil fumarate (TDF) (10, 11). In selected patients with CHB, adding pegylated interferon (Peg-IFN) to antiviral therapy may enhance HBsAg decline and increase the likelihood of HBsAg loss (12, 13). However, Peg-IFN therapy is associated with systemic adverse effects and may be accompanied by changes in appetite, body weight, thyroid function, and lipid metabolism (14–16). These effects provide a clinical rationale for evaluating longitudinal changes in 25(OH)D and related metabolic markers after Peg-IFN was added to ongoing TAF therapy.

Seasonality is an important methodological consideration in vitamin D research. Serum 25(OH)D concentrations vary substantially with sunlight exposure and season, and previous studies have modelled this cyclic pattern using sine and cosine terms or cosinor methods (17, 18). Without seasonal adjustment, an apparent longitudinal change in 25(OH)D may partly reflect the calendar timing of sampling. The primary objective of this study was therefore to characterize the 48-week trajectory of 25(OH)D during Peg-IFN add-on to ongoing TAF therapy and to assess whether the observed trajectory remained evident after seasonal adjustment. The secondary objectives were to evaluate changes in serum calcium, glycemic and lipid parameters, liver function, and measures of hepatic steatosis and fibrosis, and to explore the relationship between changes in 25(OH)D and HBsAg decline or loss.

## Methods

### Study design and participants

This single-center retrospective longitudinal cohort study used routinely collected clinical data from the hepatology clinic of West China Hospital, Sichuan University, and was reported in accordance with the STROBE statement (19). CHB was diagnosed according to international guidelines (1, 2) and defined as documented HBsAg positivity for at least 6 months. Consecutive adults aged ≥18 years who initiated Peg-IFN add-on therapy between June 2022 and November 2023 were identified from electronic medical records. Eligible patients were receiving TAF monotherapy at Peg-IFN initiation and had no contraindication to Peg-IFN. The decision to add Peg-IFN followed individualized assessment by the treating physicians; no single baseline HBsAg threshold was uniformly applied. The specific considerations underlying individual treatment decisions were not systematically recorded and could not be reconstructed consistently. Patients with HCV, HDV, or HIV coinfection, decompensated cirrhosis, hepatocellular carcinoma, pregnancy, or another contraindication to Peg-IFN were excluded.

Of 203 identified patients, 13 discontinued Peg-IFN at their own request because of adverse effects and were subsequently lost to follow-up. These patients contributed baseline data only and had no evaluable scheduled post-baseline measurements for this cohort analysis. Specific adverse-effect categories were not systematically retained. The remaining 190 patients constituted the longitudinal cohort, with assessments assigned to baseline and nominal weeks 12, 24, 36, and 48. Completion of follow-up did not imply complete testing of every secondary outcome. Follow-up continued through December 2024.

### Treatment and follow-up

At Peg-IFN initiation, all patients were receiving TAF monotherapy at 25 mg orally once daily. Exact TAF initiation dates were not consistently recorded, so individual pretreatment durations could not be reliably summarized. No other antiviral regimen was documented in the available pretreatment records. Peginterferon alfa-2b (PegBeron, Xiamen Amoytop Biotech Co., Ltd., Xiamen, China) was added at 180 μg by subcutaneous injection once weekly for a planned 48-week course. Treatment administration records confirmed that the 190 longitudinal-cohort patients received the planned course without documented dose reduction, temporary interruption, or premature Peg-IFN discontinuation. No TAF dose modification, interruption, or switching was documented during follow-up.

### Laboratory and fibroScan measurements

Total serum 25(OH)D (D2 plus D3) was measured using a competitive enzyme-linked immunosorbent assay (IDS 25-Hydroxy Vitamin D EIA; Immunodiagnostic Systems Ltd., Boldon, UK), with an analytical measurement range of 16.3–250 nmol/L. The assay-specific adult reference interval reported by the clinical laboratory was 47.7–144 nmol/L. This interval was used for routine laboratory reporting and was not derived from the present cohort; values below 47.7 nmol/L were described as below the local laboratory reference limit, not as a universal diagnosis of vitamin D deficiency. For comparison with historical literature (3), additional descriptive bands were <50, 50 to <75, and ≥75 nmol/L; these were not treated as universal diagnostic or treatment thresholds. Serum HBsAg was quantified using the Elecsys HBsAg II Quant assay on a cobas e immunoassay platform (Roche Diagnostics GmbH, Mannheim, Germany). The lower reporting limit was 0.05 IU/mL; samples above the direct analytical range were diluted and retested according to the manufacturer's instructions. Serum HBV DNA was quantified by real-time polymerase chain reaction using the COBAS AmpliPrep/COBAS TaqMan HBV Test, version 2.0 (Roche Molecular Systems, Branchburg, NJ, USA), with a linear range of 20 to 1.7 × 10^8^ IU/mL. Archived reports also contained thresholds of <10, <20, or <100 IU/mL; these historical reporting thresholds were retained individually and were not assumed to define the current platform's analytical range. Measurements were performed in the accredited clinical laboratory of West China Hospital using the same assay methods across visits. Glucose and lipid samples were obtained after an overnight fast. Calibration and routine internal quality control followed the manufacturers' procedures. Study-period coefficients of variation, formal local lower-limit-of-quantification validation, and reagent-lot histories were not available in the retrospective records; use of an identical reagent lot throughout the study could not be established. Calcium was uncorrected total serum calcium. Concurrent albumin and ionized calcium were unavailable, so albumin-adjusted calcium was not calculated. FibroScan examinations were performed by two experienced operators according to the manufacturer's standard procedure. Examinations included at least 10 valid measurements, and LSM was considered reliable when the IQR/median ratio was ≤30%. No research-specific blinding to visit time was documented. Examinations were obtained during routine clinical care and were not mandated at every nominal visit.

### Variables and outcomes

The primary outcome was longitudinal change in serum 25(OH)D from baseline through nominal week 48. Secondary outcomes included uncorrected total serum calcium, glucose, uric acid, triglycerides, total cholesterol, high-density lipoprotein cholesterol (HDL-C), low-density lipoprotein cholesterol (LDL-C), alanine aminotransferase (ALT), aspartate aminotransferase (AST), controlled attenuation parameter (CAP, dB/m), and liver stiffness measurement (LSM, kPa). Virological variables included HBV DNA and HBsAg, analyzed on the log10 scale where appropriate. Exploratory week-48 endpoints were an HBsAg decline of at least 1 log10 IU/mL from baseline and visit-specific HBsAg loss, defined strictly as an original report of <0.05 IU/mL. An exact result of 0.05 IU/mL did not meet the loss definition. Repeated HBsAg loss at weeks 36 and 48, first observed loss at week 48, and reappearance of a result ≥0.05 IU/mL after an earlier below-limit result were evaluated separately. No samples beyond week 48 were available to establish durable post-treatment loss. Information on vitamin D or calcium supplementation, lipid-modifying or thyroid medications, dietary intake, lifestyle counseling, individual sunlight exposure, body weight, physical activity, and treatment-related symptoms was not systematically recorded and was therefore not included in adjusted analyses.

### Seasonal variables

Calendar month and day of year (DOY) were derived from recorded sampling dates. Following harmonic regression and cosinor approaches (17, 18, 20), annual seasonality was represented by sin(2π × DOY/365.25) and cos(2π × DOY/365.25), entered simultaneously as fixed effects. A second-harmonic sensitivity model added sin(4π × DOY/365.25) and cos(4π × DOY/365.25). Seasonal amplitude and the fitted peak day were derived from the annual coefficients. The fitted seasonal component describes this cohort conditional on the model; it is not a measurement of individual ultraviolet exposure.

### Statistical analysis

Continuous variables were summarized as medians with interquartile ranges (IQRs), and categorical variables as counts and percentages. Available baseline-to-week-48 pairs were compared using two-sided Wilcoxon signed-rank tests, with the asymptotic calculation used because of ties. Baseline and week-48 summaries in paired tables refer to the same paired subset. Changes in the proportions below 47.7 and 50 nmol/L were tested using exact McNemar tests based on discordant pairs. Baseline characteristics of the 190 retained and 13 non-retained patients were compared using Wilcoxon rank-sum or Fisher's exact tests; absolute standardized mean differences were also reported.

Original comparator symbols were retained separately from numerical concentrations. In reporting-limit continuous analyses, HBsAg results reported as <0.05 IU/mL were assigned 0.05 IU/mL before log10 transformation, giving a boundary value of −1.30 log10 IU/mL. Half-limit sensitivity analyses assigned 0.025 IU/mL only to below-limit reports, while exact values of 0.05 IU/mL remained unchanged. Binary HBsAg-loss endpoints were defined using the original laboratory report of <0.05 IU/mL. HBV DNA reports of <10, <20, or <100 IU/mL were represented by their respective recorded thresholds before log10 transformation.

The primary longitudinal analysis of 25(OH)D used a linear mixed-effects model with categorical nominal visit time, baseline as reference, and a patient-specific random intercept. Fixed effects comprised visit time, annual seasonal sine and cosine terms based on recorded sampling dates, age, and sex. The model estimated separate differences from baseline at weeks 12, 24, 36, and 48 without imposing a linear trajectory. A continuous-time model in nominal 12-week units was retained as a secondary summary of the average trend; four times its time coefficient is a scaled 48-week linear trend, not the categorical week-48 contrast. Linear, quadratic, and categorical fixed time effects were compared using likelihood-ratio tests and Akaike information criteria (AIC) from maximum-likelihood fits to the same observations. Final mixed-model coefficients were estimated by restricted maximum likelihood (REML). Unadjusted, age- and sex-adjusted, and season-, age-, and sex-adjusted categorical models used the same analysis sample. A model adding a patient-specific linear random slope and intercept-slope covariance retained categorical fixed time effects and was compared with the random-intercept model using REML AIC because the fixed effects and sample were unchanged. The conditional random-intercept intraclass correlation coefficient (ICC) was defined as intercept variance divided by the sum of intercept and residual variances; it does not describe variability in individual slopes.

Timing sensitivity analyses used actual elapsed time in the continuous model, or seasonal dates calculated as baseline date plus nominal follow-up weeks in a categorical model. The latter was a scheduled-date scenario only: original recorded dates were not changed or replaced in the primary analysis. Separate categorical analyses excluded patients with any post-baseline visit more than 8 or 12 weeks from nominal timing or with unusable elapsed-time information. Leave-one-patient-out refits assessed influence on the primary categorical week-48 estimate. Baseline-tertile analyses included visit-by-tertile interactions; an Oldham-type Spearman correlation related change to the mean of baseline and week-48 25(OH)D. These exploratory checks cannot exclude regression to the mean when only one pretreatment measurement is available.

Secondary continuous outcomes were analyzed using random-intercept models with nominal continuous time, annual seasonality, age, and sex; their 48-week estimates summarize average linear trends. Outcome-specific diagnostics included residual-vs.-fitted and normal quantile–quantile plots, normalized-residual skewness, the percentage of absolute normalized residuals above 3, and Shapiro–Wilk tests. Diagnostic tests were interpreted with graphical and distributional information, not as proof that model assumptions held. Natural-log sensitivity models were fitted for positive triglyceride, ALT, AST, and LSM measurements. Exponentiated scaled time coefficients were expressed as relative percentage changes, distinct from raw-scale absolute changes. Benjamini–Hochberg false discovery rate (FDR) adjustment was applied separately across the 13 secondary paired comparisons and the 13 secondary mixed-model time effects; the primary 25(OH)D effect was not included in these families.

Six Spearman correlations formed a separate FDR family: baseline 25(OH)D vs. baseline HBsAg, week-48 25(OH)D vs. week-48 HBsAg, and concurrent changes from baseline to weeks 12, 24, 36, and 48. Logistic regression evaluated each of the two binary HBsAg endpoints. The exposure of interest was the magnitude of 25(OH)D decline per 10 nmol/L, adjusted for baseline log10 HBsAg, baseline 25(OH)D, age, sex, and baseline seasonal terms. Additional models evaluated baseline 25(OH)D continuously per 10 nmol/L or as <47.7 nmol/L, with baseline log10 HBsAg, age, sex, and baseline seasonal terms as covariates. Logistic confidence intervals were Wald intervals on the log-odds scale, exponentiated to odds ratios (ORs). The two models with 25(OH)D decline as the exposure formed an additional, separate two-test Benjamini–Hochberg family. The four other logistic models evaluating binary baseline vitamin D status or continuous baseline vitamin D, the time-varying models, and other sensitivity analyses retained nominal exploratory *P* values. Models using the 48-week 25(OH)D change evaluated concurrent association, not prospective prediction.

Time-varying random-intercept models of log10 HBsAg separated 25(OH)D into each patient's mean across model-eligible visits (between-patient component) and each visit's deviation from that mean (within-patient component), both scaled per 10 nmol/L. Models included categorical visit time, annual seasonal terms, age, and sex. Half-limit substitution and exclusion of patients with week-48 HBsAg loss were examined in sensitivity analyses. A further sensitivity analysis fitted a left-censored Gaussian marginal model for log10 HBsAg using a censoring likelihood and patient-cluster-robust sandwich standard errors (survival::survreg, Gaussian distribution, left censoring). Reports of <0.05 IU/mL were left-censored at log10(0.05); exact 0.05 IU/mL results remained observed. The model used the same categorical time, season, age, sex, and within-/between-patient 25(OH)D terms. It contained no random intercept. Its marginal Gaussian location parameter is not numerically interchangeable with the conditional random-intercept mixed-model coefficient.

Paired analyses used available pairs; mixed models used all observations with valid outcomes, sampling dates, and required covariates. No missing outcomes, dates, or covariates were imputed. Below-limit laboratory results were observed censored measurements, not missing values. Outcome availability and valid-date model inclusion were summarized separately by visit, together with the number of available visits per patient. Likelihood-based mixed-model inference assumes missingness is at random conditional on included observed information and an appropriate model. Missingness dependent on unobserved outcomes cannot be excluded.

The sample size was determined by consecutive eligible patients during the study window; no *a priori* sample-size target was specified. Statistical precision was described using 95% confidence intervals, not power calculated from the observed primary effect. For context, a Fisher-z approximation indicated that 190 patients provide approximately 80% power to detect an absolute correlation of 0.20 at a two-sided *α* of 0.05; this is not an exact calculation for a tied or censored Spearman statistic. All tests were two-sided. Analyses used R 4.6.0, nlme 3.1.169 for mixed models, survival 3.8.6 for the censored sensitivity, and base stats for paired tests, correlations, logistic regression, and FDR adjustment. Data management used readxl 1.5.0, dplyr 1.2.1, and tidyr 1.3.2; plots used ggplot2 4.0.3. Complete software information is provided in Supplementary Table S1 and the reproducibility files.

## Results

### Baseline characteristics

A total of 203 patients receiving TAF initiated Peg-IFN add-on therapy; 13 discontinued because of adverse effects and contributed baseline data only, leaving 190 in the longitudinal cohort (Figure 1). The median age was 40.0 years (IQR, 33.0–46.0); 118 patients (62.1%) were men and 38 (20.0%) were HBeAg positive. All were receiving TAF at Peg-IFN initiation, and 133 (70.0%) had baseline HBV DNA ≤100 IU/mL. Exact prior TAF duration was unavailable. Treatment administration records confirmed that all 190 patients completed the planned 48-week Peg-IFN add-on course without documented dose reduction, temporary interruption, or premature discontinuation. No TAF dose modification, interruption, or switching was documented during follow-up. Median baseline ALT and AST were 27.0 U/L (IQR, 20.0–41.8) and 25.5 U/L (IQR, 21.0–33.0), respectively. CAP and LSM were available for 173 patients, with medians of 224.0 dB/m (IQR, 202.0–260.0) and 5.50 kPa (IQR, 4.80–6.40), respectively (Table 1). A separately adjudicated baseline cirrhosis classification was not available, and LSM was not used alone to assign such a diagnosis.

**Figure 1:**
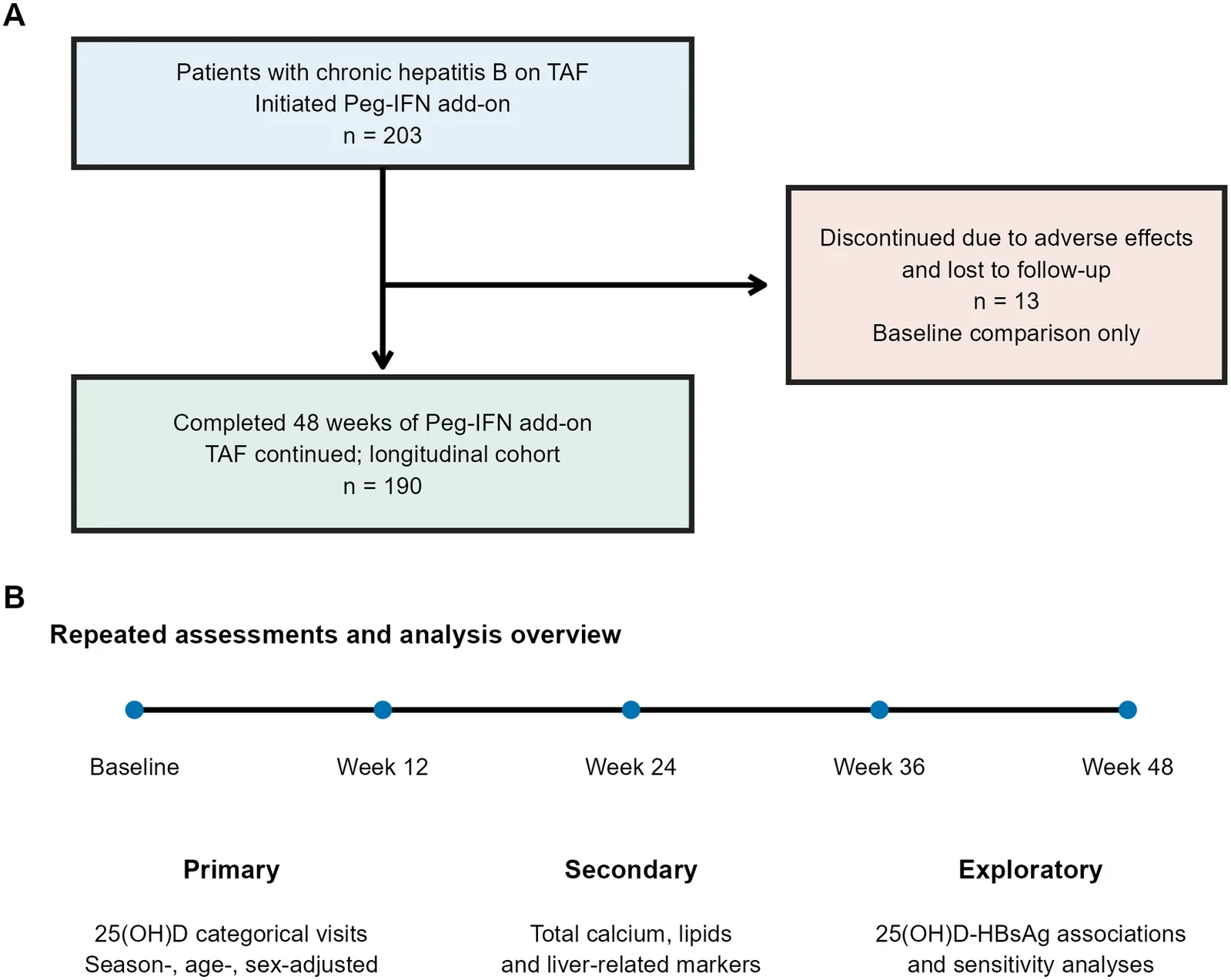
Cohort flow and analysis overview. **(A)** Cohort flow. Among 203 patients with chronic hepatitis B who initiated add-on Peg-IFN while receiving TAF, 13 discontinued Peg-IFN because of adverse effects and were subsequently lost to follow-up. The remaining 190 patients completed 48 weeks of follow-up and formed the longitudinal cohort. The 13 non-retained patients contributed only to the baseline attrition comparison. Baseline assessments were performed before Peg-IFN initiation. **(B)** Assessments conducted at baseline and weeks 12, 24, 36, and 48, and the hierarchy of primary, secondary, and exploratory analyses. TAF, tenofovir alafenamide; Peg-IFN, peginterferon alfa-2b; 25(OH)D, 25-hydroxyvitamin D; HBsAg, hepatitis B surface antigen.

**Table 1:** Baseline characteristics of the longitudinal cohort.

Characteristic	*N*	Summary
Age (years)	190	40.0 [33.0, 46.0]
25(OH)D (nmol/L)	190	68.5 [50.5, 86.4]
Total calcium (mmol/L)	190	2.28 [2.25, 2.35]
Glucose (mmol/L)	190	4.93 [4.67, 5.26]
Uric acid (µmol/L)	190	339.5 [267.3, 386.5]
Triglycerides (mmol/L)	190	1.12 [0.82, 1.76]
Total cholesterol (mmol/L)	190	4.30 [3.70, 4.88]
HDL-C (mmol/L)	190	1.31 [1.05, 1.66]
LDL-C (mmol/L)	190	2.46 [2.05, 2.97]
ALT (U/L)	190	27.0 [20.0, 41.8]
AST (U/L)	190	25.5 [21.0, 33.0]
CAP (dB/m)	173	224.0 [202.0, 260.0]
LSM (kPa)	173	5.50 [4.80, 6.40]
HBsAg (log10 IU/mL)	190	2.53 [1.66, 3.07]
HBV DNA (log10 IU/mL)	190	2.00 [2.00, 2.41]
Male sex	190	118 (62.1%)
HBeAg positive	190	38 (20.0%)
Ongoing TAF monotherapy at Peg-IFN initiation	190	190 (100.0%)
Baseline HBV DNA ≤100 IU/mL	190	133 (70.0%)

Continuous variables are median [IQR]; categorical variables are *n* (%). N is the available number of patients for each characteristic. Baseline preceded Peg-IFN initiation. CAP and LSM describe available FibroScan measurements. Exact duration of prior TAF treatment, BMI, renal function, and supplement use were not systematically available. HBeAg positivity was based on the recorded assay index of at least 1. ALT, alanine aminotransferase; AST, aspartate aminotransferase; CAP, controlled attenuation parameter; LSM, liver stiffness measurement; HDL-C, high-density lipoprotein cholesterol; LDL-C, low-density lipoprotein cholesterol; TAF, tenofovir alafenamide; Peg-IFN, pegylated interferon.

Median baseline 25(OH)D was 68.5 nmol/L (IQR, 50.5–86.4). Baseline HBV DNA and HBsAg were 2.00 log10 IU/mL (IQR, 2.00–2.41) and 2.53 log10 IU/mL (IQR, 1.66–3.07), respectively. Total calcium was 2.28 mmol/L (IQR, 2.25–2.35), and median triglycerides, total cholesterol, HDL-C, and LDL-C were 1.12, 4.30, 1.31, and 2.46 mmol/L, respectively (Table 1). The baseline HBV DNA IQR in the 149-patient paired subset was 2.00–2.34 log10 IU/mL (Table 2), reflecting the different analysis population. Compared with retained patients, the 13 non-retained patients had lower baseline HBsAg [1.84 (IQR, −1.30 to 2.72) vs. 2.53 (IQR, 1.66–3.07) log10 IU/mL; *P* = 0.034] and HBV DNA [2.00 (IQR, 1.30–2.00) vs. 2.00 (IQR, 2.00–2.41) log10 IU/mL; *P* = 0.006]. Other baseline comparisons were imprecise because of the small number of non-retained patients (Supplementary Table S2).

**Table 2:** Paired changes from baseline to week 48.

Variable	Paired n	Baseline (paired subset)	Week 48	Median change [IQR]	*P* value	FDR-adjusted P
25(OH)D (nmol/L)	190	68.5 [50.5, 86.4]	57.6 [45.7, 76.8]	−8.1 [−19.3, 4.0]	<0.001	–
25(OH)D below 47.7 nmol/L	190	40 (21.1%)	66 (34.7%)	–	<0.001	–
Total calcium (mmol/L)	190	2.28 [2.25, 2.35]	2.23 [2.18, 2.28]	−0.06 [−0.12, −0.01]	<0.001	<0.001
Glucose (mmol/L)	190	4.93 [4.67, 5.26]	4.93 [4.69, 5.23]	−0.01 [−0.25, 0.24]	0.878	0.878
Uric acid (µmol/L)	190	339.5 [267.3, 386.5]	326.5 [273.3, 377.8]	−1.5 [−26.8, 29.8]	0.815	0.878
Triglycerides (mmol/L)	190	1.12 [0.82, 1.76]	1.44 [1.08, 1.93]	0.27 [−0.10, 0.66]	<0.001	<0.001
Total cholesterol (mmol/L)	190	4.30 [3.70, 4.88]	3.97 [3.39, 4.46]	−0.29 [−0.79, 0.16]	<0.001	<0.001
HDL-C (mmol/L)	190	1.31 [1.05, 1.66]	1.12 [0.93, 1.39]	−0.18 [−0.34, −0.01]	<0.001	<0.001
LDL-C (mmol/L)	190	2.46 [2.05, 2.97]	2.19 [1.74, 2.73]	−0.19 [−0.66, 0.14]	<0.001	<0.001
ALT (U/L)	190	27.0 [20.0, 41.8]	36.0 [23.0, 59.0]	4.0 [−8.0, 26.0]	0.002	0.002
AST (U/L)	190	25.5 [21.0, 33.0]	36.0 [25.0, 50.8]	6.0 [−4.0, 22.8]	<0.001	<0.001
CAP (dB/m)	147	224.0 [204.0, 260.0]	235.0 [207.5, 261.0]	2.0 [−20.5, 29.5]	0.258	0.305
LSM (kPa)	147	5.50 [4.80, 6.50]	5.80 [4.95, 6.70]	0.30 [−0.50, 1.05]	0.016	0.020
HBsAg log10 (log10 IU/mL)	190	2.53 [1.66, 3.07]	1.06 [−1.30, 2.45]	−1.12 [−2.17, −0.42]	<0.001	<0.001
HBV DNA log10 (log10 IU/mL)	149	2.00 [2.00, 2.34]	1.30 [1.30, 2.00]	−0.67 [−0.72, 0.00]	<0.001	<0.001

Values are median [IQR] unless stated otherwise. Baseline and week-48 summaries use the same available pairs for each outcome; they may differ from the full-cohort values in Table 1. Change is week 48 minus baseline. *P* values use paired Wilcoxon signed-rank tests, except the reference-limit comparison, which uses an exact McNemar test (37 newly below; 11 no longer below). Benjamini-Hochberg adjustment was applied as one family to the 13 secondary continuous paired outcomes, excluding the primary 25(OH)D outcome. The reference-limit comparison is descriptive and was not added to that family. The laboratory interval is 47.7–144 nmol/L; crossing its lower limit is not itself evidence of clinical deterioration. For continuous HBsAg analyses, <0.05 IU/mL was assigned 0.05 before log10 transformation (−1.30 log10 IU/mL), explaining the negative lower quartile, not a negative antigen concentration. Exact 0.05 was not loss. HBV DNA below-limit results were represented by their recorded thresholds before log10 transformation; see Methods.

25(OH)D, HBsAg, and most routine biochemical outcomes had 950 measurements across the five nominal visits. Total calcium, HBV DNA, and CAP/LSM had 948, 539, and 510 measurements, respectively. After excluding observations with unusable sampling dates, 946 observations were eligible for the 25(OH)D model, 944 for calcium, 537 for HBV DNA, and 508 from 189 patients for CAP/LSM. Visit-specific availability, patient-level measurement patterns, and model-eligible observations are reported in Supplementary Tables S3A–S3C.

### Longitudinal changes in serum 25(OH)D

Observed median 25(OH)D decreased from 68.5 nmol/L (IQR, 50.5–86.4) at baseline to 62.5 at week 12, 55.0 at week 24, and 54.1 at week 36, followed by a partial rebound to 57.6 nmol/L (IQR, 45.7–76.8) at week 48 (Figure 2A; Supplementary Table S4). The median within-patient change from baseline to week 48 was −8.1 nmol/L (IQR, −19.3 to 4.0; *P* < 0.001; 190 pairs; Table 2). In the primary season-, age-, and sex-adjusted categorical model, the differences from baseline were −5.2, −8.6, −10.4, and −7.9 nmol/L at weeks 12, 24, 36, and 48, respectively. The adjusted week-48 difference was −7.9 nmol/L (95% CI, −9.8 to −6.0; *P* < 0.001; 946 observations from 190 patients; Figure 2B; Table 3). The categorical model fit better than the linear model (likelihood-ratio *χ*^2^ = 28.29, df = 3, *P* < 0.001; AIC, 7,545.83 vs. 7,568.12). Although a quadratic model also improved fit, categorical visit time was selected for primary reporting because it directly estimated differences at each scheduled visit. The secondary continuous-time model estimated an overall scaled 48-week trend of −8.3 nmol/L (95% CI, −10.2 to −6.5; *P* < 0.001; Supplementary Tables S5A, S5B).

**Figure 2:**
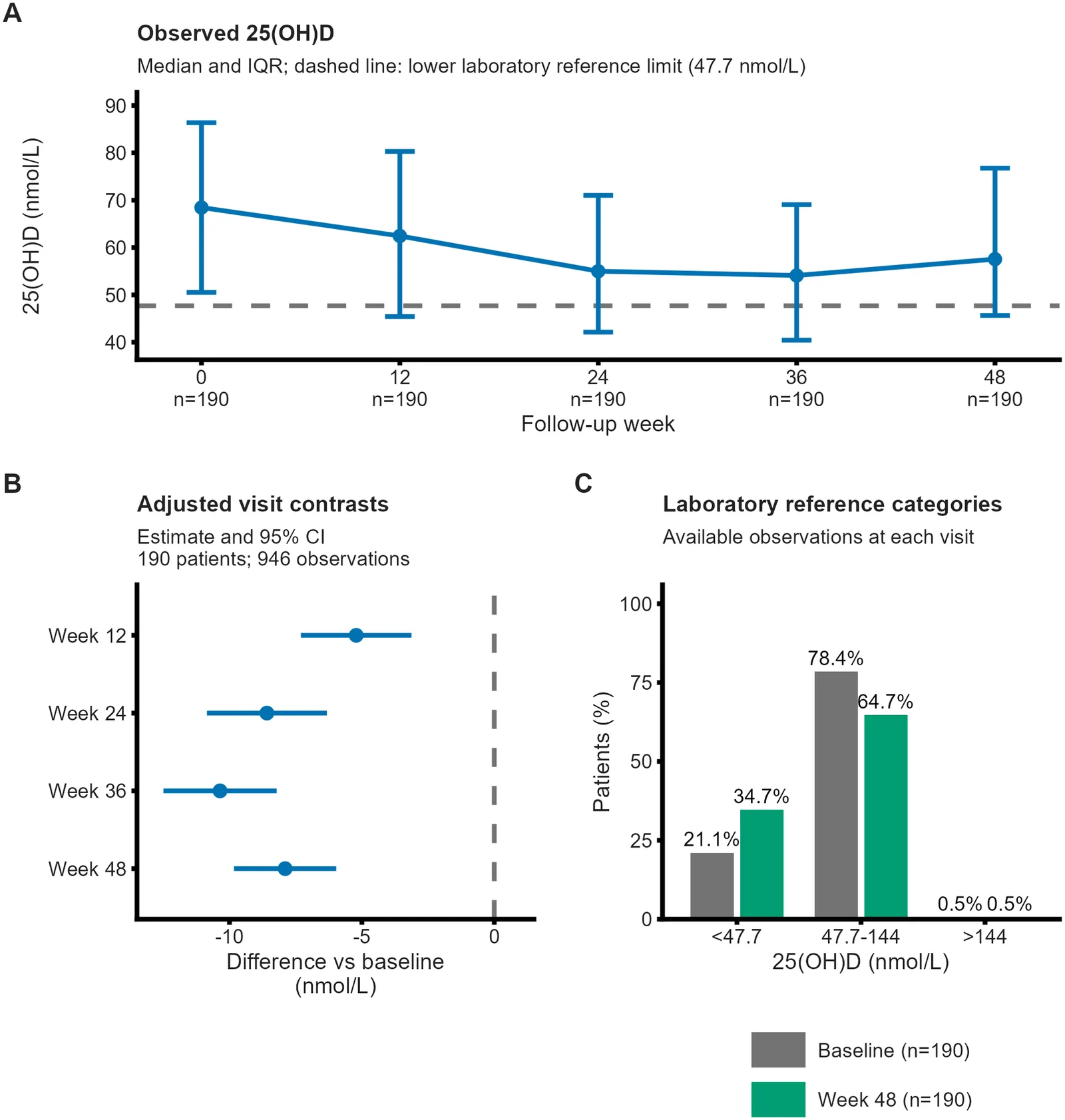
Observed and adjusted serum 25(OH)D trajectories. Baseline assessments were performed before Peg-IFN initiation; weeks 12–48 refer to follow-up after Peg-IFN was added to ongoing TAF therapy. **(A)** Observed serum 25(OH)D concentrations. Points show medians and bars show interquartile ranges; *n* = 190 at each visit. The dashed line indicates the local laboratory lower reference limit of 47.7 nmol/L. **(B)** Adjusted differences from baseline with 95% confidence intervals, estimated using a patient-specific random-intercept mixed-effects model that included categorical visit time, annual seasonal sine and cosine terms, age, and sex. Four observations were excluded because their sampling dates were unusable for seasonal adjustment, leaving 946 observations from 190 patients. Estimates represent visit-specific contrasts rather than the scaled continuous-time trend. **(C)** Proportions of patients below (<47.7 nmol/L), within (47.7–144 nmol/L), or above (>144 nmol/L) the local laboratory reference interval at baseline and week 48 (*n* = 190 at each visit). Gray bars represent baseline and green bars represent week 48. Categories are based on the local laboratory reference interval rather than clinical treatment thresholds. 25(OH)D, 25-hydroxyvitamin D; TAF, tenofovir alafenamide; Peg-IFN, peginterferon alfa-2b.

**Table 3:** Adjusted changes in laboratory and virological markers.

Outcome	Patients	Observations	Model	Estimate (95% CI)	*P* value	FDR-adjusted *P*
25(OH)D (nmol/L)	190	946	Categorical visit	−7.9 (−9.8 to −6.0)	<0.001	–
Total calcium (mmol/L)	190	944	Continuous time	−0.061 (−0.074 to −0.049)	<0.001	<0.001
Glucose (mmol/L)	190	946	Continuous time	0.01 (−0.05 to 0.06)	0.753	0.753
Uric acid (µmol/L)	190	946	Continuous time	4.1 (−2.8 to 10.9)	0.244	0.318
Triglycerides (mmol/L)	190	946	Continuous time	0.28 (0.08 to 0.48)	0.006	0.009
Total cholesterol (mmol/L)	190	946	Continuous time	−0.30 (−0.38 to −0.21)	<0.001	<0.001
HDL-C (mmol/L)	190	946	Continuous time	−0.17 (−0.21 to −0.14)	<0.001	<0.001
LDL-C (mmol/L)	190	946	Continuous time	−0.24 (−0.32 to −0.17)	<0.001	<0.001
ALT (U/L)	190	946	Continuous time	−2.6 (−11.3 to 6.0)	0.552	0.598
AST (U/L)	190	946	Continuous time	3.0 (−3.3 to 9.4)	0.351	0.415
CAP (dB/m)	189	508	Continuous time	4.5 (−1.8 to 10.8)	0.160	0.231
LSM (kPa)	189	508	Continuous time	0.50 (0.18 to 0.81)	0.002	0.004
HBsAg log10 (log10 IU/mL)	190	946	Continuous time	−1.43 (−1.56 to −1.31)	<0.001	<0.001
HBV DNA log10 (log10 IU/mL)	190	537	Continuous time	−0.86 (−1.01 to −0.71)	<0.001	<0.001

All models included a patient-specific random intercept, annual seasonal sine and cosine terms based on the recorded sampling date, age, and sex. For the primary 25(OH)D analysis, visit was categorical and the estimate is the week-48 difference from baseline. For secondary continuous outcomes, time was in 12-week units and the estimate is four times the time coefficient: an average cumulative 48-week trend, not a visit-specific contrast. Benjamini-Hochberg adjustment was applied to the 13 secondary mixed-model outcomes as a family separate from the paired tests. The primary 25(OH)D contrast was not FDR-adjusted. Missing outcomes or required covariates were not imputed. Viral log-scale models used reporting-limit substitution; HBsAg reporting-limit and censoring sensitivity analyses are reported in Supplementary Tables S9B, S9C. CI, confidence interval.

Using the local 47.7–144 nmol/L reference interval, 40/190 patients (21.1%) were below the lower limit at baseline, 149 (78.4%) were within the interval, and one (0.5%) was above it. At week 48, the corresponding counts were 66 (34.7%), 123 (64.7%), and one (0.5%). Thirty-seven patients newly fell below the lower limit and 11 recovered (exact McNemar *P* < 0.001; Figure 2C; Table 2). In the <50 nmol/L sensitivity, 47/190 (24.7%) were below the cutoff at baseline and 68/190 (35.8%) at week 48; 33 newly fell below it and 12 recovered (*P* = 0.002). The full <50, 50 to <75, and ≥75 nmol/L distributions and transitions are reported in Supplementary Tables S6A, S6B, S6D.

Individual trajectories showed substantial heterogeneity (Supplementary Figure S1). Baseline sampling covered 11 months but was concentrated from February through July (172/190, 90.5%). The fitted seasonal terms corresponded to an annual amplitude of 2.8 nmol/L and a peak near 5 June; adding a semi-annual harmonic did not improve model fit (*P* = 0.156). On the same 946 observations, the unadjusted differences from baseline were −6.2, −11.4, −12.6, and −7.8 nmol/L at weeks 12, 24, 36, and 48, respectively. The corresponding season-, age-, and sex-adjusted estimates were −5.2, −8.6, −10.4, and −7.9 nmol/L. Seasonal adjustment therefore attenuated the estimated declines at weeks 12–36 but had little influence on the week-48 estimate (Supplementary Tables S7A–S7C; Supplementary Figures S2, S3).

Sensitivity analyses supported the primary result. The random-intercept-and-slope model estimated a week-48 difference of −7.8 nmol/L (95% CI, −10.5 to −5.1), compared with −7.9 nmol/L (95% CI, −9.8 to −6.0) in the primary random-intercept model. Models based on actual elapsed time and scheduled visit dates produced estimates of −7.8 and −7.6 nmol/L, respectively. Visit-window restrictions yielded estimates of −6.1 nmol/L in the ±8-week analysis and −7.5 nmol/L in the ±12-week analysis. Thus, the estimated magnitude varied modestly with visit-timing definitions, but all estimates remained negative (Supplementary Tables S5A, S5C; Supplementary Figures S4A–C).

Across 190 leave-one-patient-out refits, the adjusted week-48 estimates ranged from −8.2 to −7.6 nmol/L, with all confidence intervals below zero (Supplementary Table S5D). Baseline-tertile analyses showed progressively larger declines at higher baseline 25(OH)D concentrations: 2.0 nmol/L (95% CI, −1.1 to 5.2), −9.1 nmol/L (95% CI, −12.2 to −6.0), and −16.7 nmol/L (95% CI, −19.9 to −13.6) in the lowest, middle, and highest tertiles, respectively. However, the Oldham-type correlation was weak (*ρ* = −0.064; *P* = 0.383; Supplementary Table S6C).

### Changes in secondary markers

Total serum calcium decreased from 2.28 mmol/L (IQR, 2.25–2.35) at baseline to 2.23 mmol/L (IQR, 2.18–2.28) at week 48, with a paired median change of −0.06 mmol/L (IQR, −0.12 to −0.01; *P* < 0.001; 190 pairs). The adjusted continuous-time 48-week trend was −0.061 mmol/L (95% CI, −0.074 to −0.049) (nominal and FDR-adjusted *P* < 0.001; 944 observations from 190 patients). Observed calcium and lipid trajectories are shown separately in Figure 3. Triglycerides increased by 0.28 mmol/L (95% CI, 0.08 to 0.48; *P* = 0.006; FDR-adjusted *P* = 0.009), while total cholesterol, HDL-C, and LDL-C changed by −0.30, −0.17, and −0.24 mmol/L, respectively (all FDR-adjusted *P* < 0.001). Glucose and uric acid had no statistically significant adjusted trends (Tables 2, 3).

**Figure 3:**
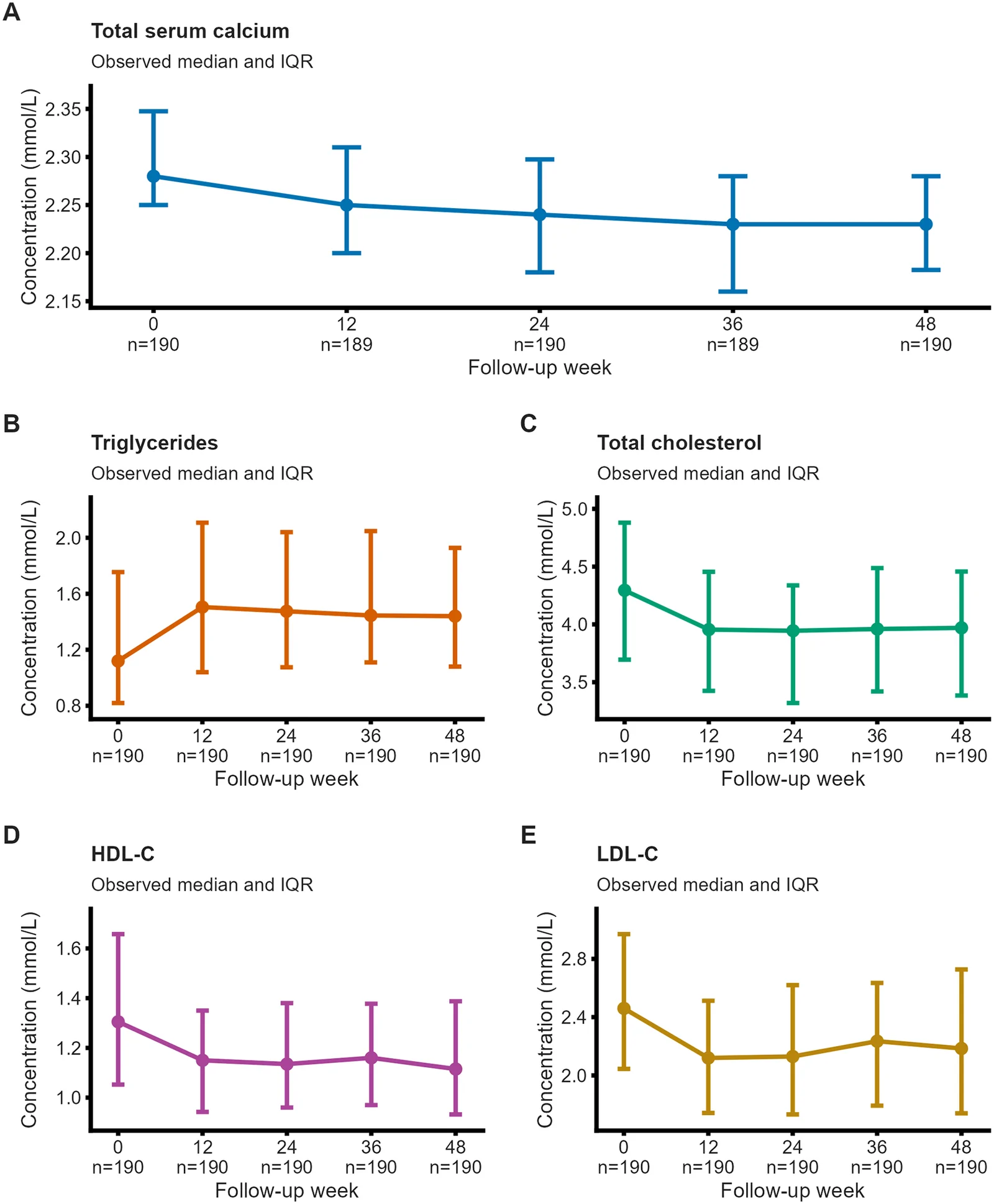
Total serum calcium and lipid trajectories. Observed trajectories of **(A)** total serum calcium, **(B)** triglycerides, **(C)** total cholesterol, **(D)** HDL-C, and **(E)** LDL-C. Points show visit medians and bars show interquartile ranges. All concentrations are reported in mmol/L, and each panel has an independent vertical scale. For total serum calcium, the available sample sizes at baseline and weeks 12, 24, 36, and 48 were 190, 189, 190, 189, and 190, respectively. For all lipid measurements, *n* = 190 at each visit. Calcium values represent total serum calcium. HDL-C, high-density lipoprotein cholesterol; LDL-C, low-density lipoprotein cholesterol.

ALT and AST increased in paired baseline-to-week-48 comparisons, whereas the adjusted continuous-time trends were −2.6 U/L (95% CI, −11.3 to 6.0; *P* = 0.552) and 3.0 U/L (95% CI, −3.3 to 9.4; *P* = 0.351), respectively. Among 147 patients with paired FibroScan measurements, median LSM increased from 5.50 to 5.80 kPa, with an adjusted trend of 0.50 kPa (95% CI, 0.18 to 0.81; FDR-adjusted *P* = 0.004). CAP did not show a significant adjusted trend. The adjusted HBV DNA trend was −0.86 log10 IU/mL (95% CI, −1.01 to −0.71; 537 observations; FDR-adjusted *P* < 0.001). Outcome-specific residual diagnostics indicated skewness for several secondary outcomes. Log-scale sensitivity analyses supported the triglyceride and LSM findings, whereas the AST inference differed between the raw- and log-scale models. These secondary results were considered exploratory (Supplementary Tables S8A, S8B; Supplementary Figure S5).

### Associations between 25(OH)D and HBsAg responses

All 190 patients had baseline HBsAg ≥0.05 IU/mL, including two exact reported values of 0.05 IU/mL; none met the strict baseline loss definition. At week 48, 104/190 patients (54.7%) had an HBsAg decline of at least 1 log10 IU/mL and 50/190 (26.3%) had an original report of <0.05 IU/mL. Of those 50 patients, 41 also had below-limit results at week 36; the remaining nine did not have consecutive week-36/week-48 loss. Only 7 had their first observed loss at week 48. Across the cohort, 3 patients had a subsequent result ≥0.05 IU/mL after an earlier below-limit result. These findings represent within-study, visit-specific patterns rather than established persistent post-treatment HBsAg loss (Table 4; Supplementary Table S9).

**Table 4:** Exploratory associations between 25(OH)D and HBsAg.

Analysis	Measure	Patients	Observations/events	Estimate (95% CI)	*P* value	FDR-adjusted *P*
48-week changes in 25(OH)D and HBsAg	Spearman rho	190	–	0.150	0.039	0.117
HBsAg decline ≥1log10	OR per 10 nmol/L decline	190	104 events	1.07 (0.88 to 1.31)	0.478	0.651
HBsAg loss	OR per 10 nmol/L decline	190	50 events	1.06 (0.81 to 1.39)	0.651	0.651
Within-patient concurrent 25(OH)D	Beta per 10 nmol/L	190	946 observations	0.068 (0.020 to 0.116)	0.006	–
Between-patient mean 25(OH)D	Beta per 10 nmol/L	190	946 observations	0.018 (−0.088 to 0.123)	0.746	–

Change-correlation multiplicity adjustment covers six correlations, all reported in Supplementary Table S9A. Logistic models adjusted for baseline HBsAg, baseline 25(OH)D, age, sex, and baseline seasonal sine/cosine terms; their two endpoints constitute a separate Benjamini-Hochberg family. A positive decline exposure means a larger 25(OH)D decrease. Time-varying models included within-patient deviations and between-patient mean 25(OH)D, categorical visit, concurrent season, age, sex, and a patient-specific random intercept. Beta is the difference in log10 HBsAg per 10 nmol/L higher 25(OH)D. Time-varying *P* values are nominal exploratory values, not part of the six-correlation family. Concurrent association does not establish prospective prediction or causality. HBsAg loss requires a report of <0.05 IU/mL; an exact 0.05 is not loss.

Among the six correlation analyses, correlations involving baseline and week-48 concentrations and changes at weeks 12 and 24 were not statistically significant. Changes in 25(OH)D and HBsAg at weeks 36 and 48 showed weak positive correlations (*ρ* = 0.154 and 0.150; nominal *P* = 0.034 and 0.039, respectively), but neither remained significant after FDR adjustment (both adjusted *P* = 0.117; Supplementary Table S9A; Supplementary Figure S6). The week-48 correlation remained weak after half-limit substitution (*ρ* = 0.154; *P* = 0.034) and after excluding patients with week-48 HBsAg loss (*ρ* = 0.101; *P* = 0.237; *n* = 140; Supplementary Table S9B). In adjusted logistic models, a 10 nmol/L greater decline in 25(OH)D was not significantly associated with either an HBsAg decline of at least 1 log10 IU/mL (OR, 1.07; 95% CI, 0.88–1.31; *P* = 0.478) or HBsAg loss (OR, 1.06; 95% CI, 0.81–1.39; *P* = 0.651; Table 4). Baseline 25(OH)D below 47.7 nmol/L was also not significantly associated with either endpoint (Supplementary Table S10).

In the categorical time-varying mixed-effects model, a 10 nmol/L higher within-patient 25(OH)D concentration was associated with 0.068 log10 IU/mL higher concurrent HBsAg (95% CI, 0.020–0.116; *P* = 0.006), whereas the between-patient coefficient was not significant (0.018 log10 IU/mL; 95% CI, −0.088 to 0.123; *P* = 0.746; Table 4). The half-limit analysis produced a similar within-patient estimate of 0.073 log10 IU/mL (95% CI, 0.022–0.124; *P* = 0.005). In the left-censored marginal sensitivity analysis, the within-patient coefficient was 0.075 log10 IU/mL (95% CI, 0.003–0.147; *P* = 0.041), while the between-patient coefficient remained non-significant (Supplementary Table S9C).

## Discussion

In this longitudinal cohort, serum 25(OH)D declined through nominal week 36 and partially rebounded by week 48 after Peg-IFN was added to ongoing TAF therapy. The primary categorical model estimated an adjusted week-48 difference of −7.9 nmol/L (95% CI, −9.8 to −6.0). This change was observed during add-on therapy but cannot be attributed specifically to Peg-IFN, ongoing TAF, or their combination because no concurrent comparator was available. The decline remained evident across the performed timing and random-effects sensitivities, although its magnitude and uncertainty varied. The magnitude of 25(OH)D decline was not independently associated with the binary HBsAg endpoints; a small positive concurrent within-patient association with repeated HBsAg was observed and should be distinguished from those endpoint analyses.

Previous studies have suggested that vitamin D status may be associated with the virological and clinical characteristics of chronic HBV infection. Farnik et al. reported that lower serum 25(OH)D levels were associated with higher levels of HBV replication (5). Subsequent observational studies and meta-analyses have also linked lower vitamin D levels to higher HBV DNA levels or HBV-related liver disease (7–9). However, most of the available evidence comes from cross-sectional studies or baseline measurements and therefore primarily reflects differences between patients. These studies cannot determine whether 25(OH)D changes longitudinally within the same patient during treatment. Moreover, without adequate adjustment for the timing of blood sampling, observed differences in vitamin D levels may be influenced by seasonal variation in sunlight exposure. Repeated measurements in the present study extend previous evidence by demonstrating a non-linear within-patient trajectory that remained evident after seasonal adjustment. However, the absence of a comparator means that this trajectory may reflect treatment-period changes in inflammation, behavior, sunlight exposure, nutritional intake, disease activity, or other time-varying factors rather than a direct pharmacological effect. These findings also suggest that a single baseline measurement may not adequately represent vitamin D status throughout follow-up.

Uncorrected total serum calcium decreased modestly during follow-up. Vitamin D contributes to calcium homeostasis, and prior work links seasonal 25(OH)D variation to mineral and bone markers (3, 20). However, parallel changes in total 25(OH)D and total calcium do not establish a direct relationship or a specific mineral-metabolism mechanism. Albumin, ionized calcium, parathyroid hormone, phosphate, bone-turnover markers, and bone density were unavailable. The observed total-calcium decline therefore cannot be assumed to represent reduced physiologically active calcium. TAF's more favorable renal and bone safety profile than TDF (10, 11) does not establish an explanation for these changes during add-on therapy. The clinical importance of the 25(OH)D decline also remains uncertain: crossing the local 47.7 nmol/L boundary may reflect a small shift near an assay-specific cutoff, rather than clinically important deterioration. Symptoms were not systematically recorded, so absence of symptomatic hypocalcaemia cannot be inferred.

Peg-IFN therapy can promote HBsAg decline or loss in selected patients (12, 13), making the relationship between vitamin D and HBsAg worthy of exploration. Prior CHB evidence has also examined baseline vitamin D in relation to treatment outcomes (6). In this cohort, neither baseline 25(OH)D status nor its 48-week decline showed a statistically supported adjusted association with the binary HBsAg endpoints, and the simple change correlation did not survive correction across the six tests. In contrast, within-patient 25(OH)D deviations were positively associated with concurrent HBsAg, while between-patient mean 25(OH)D was not. The censored marginal analysis retained a positive within-patient coefficient but did not replicate the conditional random-intercept estimand. This pattern is consistent with synchronous changes, not evidence that higher vitamin D causes higher HBsAg or that vitamin D decline promotes HBsAg loss. Shared time trends, residual time-varying confounding, and distributional assumptions remain relevant despite categorical-time and seasonal adjustment. Substitution-based and fully left-censored models address the reporting floor differently; agreement in direction does not validate the causal interpretation. These analyses were not prospective prediction studies. A bystander interpretation remains one possible explanation (21), while wide endpoint confidence intervals preclude a definitive claim of no biological association.

The assay measured total circulating 25(OH)D, not free or bioavailable 25(OH)D. Waldron et al. observed decreases in total 25(OH)D and vitamin D-binding protein during the acute inflammatory response following orthopedic surgery (22). Schwartz et al. found that calculated and directly measured free 25(OH)D differed across clinical populations, including patients with cirrhosis (23). These findings highlight the importance of inflammatory state and binding-protein context when interpreting total 25(OH)D, but do not establish a mechanism in this CHB cohort. Albumin, vitamin D-binding protein, free 25(OH)D, and inflammatory markers were not measured together here. Consequently, a decline in total 25(OH)D cannot by itself establish depletion of vitamin D stores or altered physiologically available vitamin D.

The LSM increase was small in absolute magnitude and does not establish fibrosis progression. Neither histological progression nor stable inflammatory activity was demonstrated, and routine-care examination availability was incomplete and uneven. The paired endpoint tests and continuous-time models address different aspects of change, with different samples, covariate adjustment, and use of intermediate visits. Together with skewness and scale sensitivity, these differences limit conclusions from the ALT and AST findings. Likewise, rising triglycerides alongside declining total cholesterol, LDL-C, and HDL-C represent a divergent lipid pattern, not uniform metabolic improvement or deterioration. Unrecorded diet, weight change, and concomitant medications further limit its interpretation.

This study has several strengths. Repeated 25(OH)D measurements in 190 patients allowed the non-linear trajectory to be characterized beyond an endpoint comparison. Seasonal terms used recorded sampling dates, with unadjusted, age-/sex-adjusted, and fully adjusted estimates compared on the same observations. The random-slope model explicitly demonstrated that allowing individual trajectories to vary improved fit and widened uncertainty; the random-intercept model was not preferred on goodness of fit alone. The observed decline was not attributable to a single influential patient. Nonetheless, the narrower timing subset showed a smaller estimated decline, and the recorded-date discrepancies could not be resolved as verified date corrections. These sensitivities support the direction of the trajectory without establishing identical magnitude across specifications.

This study also has several limitations. First, the observational single-center design and absence of an untreated, TAF-only, or Peg-IFN-only concurrent comparator preclude treatment-specific causal attribution. Seasonal adjustment does not account for individual changes in supplementation, diet, sunlight exposure, body weight, physical activity, inflammation, disease activity, lipid-modifying or thyroid medications, or other time-varying clinical factors. Baseline BMI, serum creatinine, estimated glomerular filtration rate, albumin, vitamin D-binding protein, and relevant endocrine and bone measurements were not systematically available. Residual confounding and uncertainty about the clinical meaning of total 25(OH)D and total calcium therefore remain.

Second, treatment selection was individualized rather than based on a uniformly applied HBsAg cutoff. The specific clinical considerations underlying individual treatment decisions could not be reconstructed systematically. Exact prior TAF duration was unavailable, and generalizability to the broader TAF-treated CHB population is limited. Third, the 13 non-retained patients differed in baseline HBsAg and HBV DNA distributions, so attrition bias cannot be excluded, particularly for HBsAg endpoints. Secondary-outcome missingness was strongly visit-dependent; a missing-not-at-random mechanism remains possible. Residual diagnostics also indicated departures from Gaussian assumptions, and sensitivity analyses do not resolve every outcome-specific distributional concern.

Fourth, declines were larger among patients with higher baseline 25(OH)D, making regression to the mean and baseline-dependent variation plausible contributors. A non-significant Oldham-type check does not exclude these explanations; only one pretreatment measurement was available. Fifth, HBsAg association analyses were exploratory and concurrent. The left-censored marginal sensitivity assumes a Gaussian latent outcome and has a different estimand from the conditional mixed model. Finally, the absence of post-week-48 samples prevents assessment of persistent post-treatment HBsAg loss or persistence of the vitamin D trajectory.

Sampling season should be considered when interpreting serial 25(OH)D results, but these findings do not establish a clinical indication for universal vitamin D or calcium monitoring during Peg-IFN add-on therapy. Prospective controlled studies incorporating supplement use, sunlight exposure, body composition, total and free 25(OH)D, albumin, vitamin D-binding protein, inflammatory markers, parathyroid hormone, phosphate, and clinically relevant bone outcomes are needed. Follow-up after Peg-IFN cessation, controlled evaluation of supplementation, and replication across centers and latitudes would help establish persistence and generalizability.

## Conclusions

Serum 25(OH)D showed a non-linear decline during 48 weeks of Peg-IFN add-on to ongoing TAF therapy, with a nadir at nominal week 36 and partial rebound by week 48. The primary categorical week-48 difference remained evident after adjustment (−7.9 nmol/L (95% CI, −9.8 to −6.0)), but the observational design does not identify a treatment-specific cause. A small concurrent within-patient 25(OH)D–HBsAg association was observed, whereas the adjusted binary endpoint associations were not statistically supported; neither finding establishes prospective prediction or causality. The modest decline in uncorrected total calcium does not establish altered physiologically active calcium or mineral metabolism. Prospective controlled studies incorporating albumin-adjusted or ionized calcium and clinically relevant mineral and bone outcomes are needed to determine the clinical significance of these laboratory changes.

## Data Availability

De-identified data may be available from the corresponding author upon reasonable request, subject to approval by the relevant ethics committee and institutional data-access requirements.
