# Supplementary material for: Season-adjusted longitudinal changes in serum 25-hydroxyvitamin D during pegylated interferon add-on to ongoing tenofovir alafenamide therapy in chronic hepatitis B: a real-world cohort study
Source: Front Med (Lausanne). 2026 Sep 30;13:1976727. doi: 10.3389/fmed.2026.1976727 (PMC13661473; doi:10.3389/fmed.2026.1976727)
Supplement: Supplementary file 1 [file Datasheet1.docx]

**Supplementary Figure S1. Individual 25(OH)D trajectories and paired changes**

(A) Individual serum 25(OH)D trajectories from baseline to week 48. Grey lines represent individual patients, and the blue points and line represent visit medians. All 190 patients had measurements at each visit. The dashed line indicates the local laboratory lower reference limit of 47.7 nmol/L. (B) Distribution of within-patient changes from baseline to week 48 (n = 190). The median change was −8.1 nmol/L (IQR, −19.3 to 4.0). The orange line indicates the median change, and the dashed line indicates no change.

**Supplementary Figure S2. Baseline sampling months and annual seasonal component**

(A) Distribution of baseline sampling months among 190 patients. (B) Fitted annual seasonal component of serum 25(OH)D from a random-intercept mixed-effects model including categorical visit time, annual sine and cosine terms, age, and sex (190 patients; 946 observations). The dashed line indicates zero seasonal contribution.

**Supplementary Figure S3. Unadjusted and adjusted categorical visit contrasts**

Visit-specific differences in serum 25(OH)D (nmol/L) from baseline in three categorical visit-time mixed-effects models: unadjusted (grey circles), adjusted for age and sex (green squares), and adjusted for season, age, and sex (blue triangles). All models included a patient-specific random intercept and used the same 946 observations from 190 patients. Points show estimates, and error bars show 95% confidence intervals. The dashed line indicates no difference from baseline.

**Supplementary Figure S4. Sensitivity analyses for visit timing and random-effects structure**

(A) Adjusted week-48 differences from baseline from the categorical visit-time model and sensitivity analyses using scheduled seasonal dates or restricting deviations from nominal visit weeks. Sample sizes are shown for each analysis. (B) Adjusted week-48 differences from random-intercept and random-intercept-plus-linear-random-slope models; AIC values are provided for model comparison. (C) Adjusted 48-week changes estimated using continuous nominal and actual follow-up time. All models were adjusted for seasonality, age, and sex. Points indicate estimates, horizontal bars indicate 95% confidence intervals, and dashed lines indicate no difference from baseline. AIC, Akaike information criterion.

**Supplementary Figure S5. Diagnostics for the adjusted categorical visit-time model**

Diagnostic plots for the categorical visit-time mixed-effects model adjusted for seasonality, age, and sex (190 patients; 946 observations). The model included a patient-specific random intercept. (A) Normalized residuals versus fitted serum 25(OH)D concentrations; the dashed line indicates zero residual. (B) Normal Q-Q plot of the normalized residuals; the orange reference line connects the first and third quartiles.

**Supplementary Figure S6. Exploratory association between 48-week changes in 25(OH)D and HBsAg**

Association between changes from baseline to week 48 in serum 25(OH)D and log10-transformed HBsAg (n = 190). Each point represents one patient. Blue circles indicate exact HBsAg measurements at both visits (n = 140), and orange triangles indicate an HBsAg result below the assay limit at week 48 (<0.05 IU/mL; n = 50). Spearman ρ = 0.150. Dashed lines indicate no change. Values below the assay limit were handled as described in the Methods. HBsAg, hepatitis B surface antigen.
