# Supplementary material for: Season-adjusted longitudinal changes in serum 25-hydroxyvitamin D during pegylated interferon add-on to ongoing tenofovir alafenamide therapy in chronic hepatitis B: a real-world cohort study
Source: Front Med (Lausanne). 2026 Sep 30;13:1976727. doi: 10.3389/fmed.2026.1976727 (PMC13661473; doi:10.3389/fmed.2026.1976727)
Supplement: Supplementary file 2 [file Datasheet2.docx]

**Supplementary Table S1. Software and R package versions used in the analyses**

| **Software or package** | **Version** |
| --- | --- |
| R | 4.6.0 |
| readxl | 1.5.0 |
| writexl | 1.5.4 |
| dplyr | 1.2.1 |
| tidyr | 1.3.2 |
| ggplot2 | 4.0.3 |
| nlme | 3.1.169 |
| scales | 1.4.0 |
| survival | 3.8.6 |

Note: Mixed-effects models were fitted using nlme, and the left-censored sensitivity analysis was performed using survival. Other statistical tests and false discovery rate adjustment used base R functions.

**Supplementary Table S2. Baseline characteristics of retained participants and participants lost to follow-up**

| **Variable** | **Retained participants** | | **Participants lost to follow-up** | | **P value** | **Absolute SMD** |
| --- | --- | --- | --- | --- | --- | --- |
|  | **N** | **Summary** | **N** | **Summary** |  |  |
| Age, years | 190 | 40.0 [33.0, 46.0] | 13 | 46.0 [40.0, 48.0] | 0.128 | 0.419 |
| 25(OH)D, nmol/L | 190 | 68.5 [50.5, 86.4] | 13 | 59.4 [55.2, 77.9] | 0.470 | 0.255 |
| HBsAg, log10 IU/mL | 190 | 2.53 [1.66, 3.07] | 13 | 1.84 [−1.30, 2.72] | 0.034 | 0.726 |
| HBV DNA, log10 IU/mL | 190 | 2.00 [2.00, 2.41] | 13 | 2.00 [1.30, 2.00] | 0.006 | 0.789 |
| ALT, U/L | 190 | 27.0 [20.0, 41.8] | 13 | 25.0 [19.0, 40.0] | 0.783 | 0.123 |
| AST, U/L | 190 | 25.5 [21.0, 33.0] | 13 | 23.0 [18.0, 26.0] | 0.144 | 0.325 |
| LSM, kPa | 173 | 5.50 [4.80, 6.40] | 13 | 5.90 [5.10, 6.60] | 0.304 | 0.456 |
| CAP, dB/m | 173 | 224.0 [202.0, 260.0] | 13 | 246.0 [221.0, 277.0] | 0.105 | 0.395 |
| Male sex | 190 | 118/190 (62.1%) | 13 | 11/13 (84.6%) | 0.139 | 0.527 |
| HBeAg positive | 190 | 38/190 (20.0%) | 13 | 0/13 (0.0%) | 0.133 | 0.707 |

Note: Values are median [IQR] or n/N (%). P values were calculated using Wilcoxon rank-sum tests for continuous variables and Fisher’s exact tests for categorical variables. HBeAg positivity was defined as a recorded assay index ≥1. SMD, standardized mean difference.

**Supplementary Table S3A. Outcome availability at each scheduled visit**

| **Outcome** | **Baseline** | **Week 12** | **Week 24** | **Week 36** | **Week 48** |
| --- | --- | --- | --- | --- | --- |
| 25(OH)D | 190/190 (100.0%) | 190/190 (100.0%) | 190/190 (100.0%) | 190/190 (100.0%) | 190/190 (100.0%) |
| Total calcium | 190/190 (100.0%) | 189/190 (99.5%) | 190/190 (100.0%) | 189/190 (99.5%) | 190/190 (100.0%) |
| Glucose | 190/190 (100.0%) | 190/190 (100.0%) | 190/190 (100.0%) | 190/190 (100.0%) | 190/190 (100.0%) |
| Uric acid | 190/190 (100.0%) | 190/190 (100.0%) | 190/190 (100.0%) | 190/190 (100.0%) | 190/190 (100.0%) |
| Triglycerides | 190/190 (100.0%) | 190/190 (100.0%) | 190/190 (100.0%) | 190/190 (100.0%) | 190/190 (100.0%) |
| Total cholesterol | 190/190 (100.0%) | 190/190 (100.0%) | 190/190 (100.0%) | 190/190 (100.0%) | 190/190 (100.0%) |
| HDL-C | 190/190 (100.0%) | 190/190 (100.0%) | 190/190 (100.0%) | 190/190 (100.0%) | 190/190 (100.0%) |
| LDL-C | 190/190 (100.0%) | 190/190 (100.0%) | 190/190 (100.0%) | 190/190 (100.0%) | 190/190 (100.0%) |
| ALT | 190/190 (100.0%) | 190/190 (100.0%) | 190/190 (100.0%) | 190/190 (100.0%) | 190/190 (100.0%) |
| AST | 190/190 (100.0%) | 190/190 (100.0%) | 190/190 (100.0%) | 190/190 (100.0%) | 190/190 (100.0%) |
| CAP | 173/190 (91.1%) | 18/190 (9.5%) | 136/190 (71.6%) | 25/190 (13.2%) | 158/190 (83.2%) |
| LSM | 173/190 (91.1%) | 18/190 (9.5%) | 136/190 (71.6%) | 25/190 (13.2%) | 158/190 (83.2%) |
| HBsAg log10 | 190/190 (100.0%) | 190/190 (100.0%) | 190/190 (100.0%) | 190/190 (100.0%) | 190/190 (100.0%) |
| HBV DNA log10 | 190/190 (100.0%) | 38/190 (20.0%) | 133/190 (70.0%) | 29/190 (15.3%) | 149/190 (78.4%) |

Note: Values are available n/N (%), with N = 190 at each visit. No missing values were imputed. CAP, controlled attenuation parameter; LSM, liver stiffness measurement; HBsAg, hepatitis B surface antigen; HBV, hepatitis B virus.

**Supplementary Table S3B. Number of available measurements per participant by outcome**

| **Outcome** | **Number of available measurements** | | | | | |
| --- | --- | --- | --- | --- | --- | --- |
|  | **0** | **1** | **2** | **3** | **4** | **5** |
| 25(OH)D | 0 | 0 | 0 | 0 | 0 | 190 |
| ALT | 0 | 0 | 0 | 0 | 0 | 190 |
| AST | 0 | 0 | 0 | 0 | 0 | 190 |
| CAP | 1 | 5 | 54 | 123 | 7 | 0 |
| Glucose | 0 | 0 | 0 | 0 | 0 | 190 |
| HBV DNA log10 | 0 | 8 | 42 | 114 | 25 | 1 |
| HBsAg log10 | 0 | 0 | 0 | 0 | 0 | 190 |
| HDL-C | 0 | 0 | 0 | 0 | 0 | 190 |
| LDL-C | 0 | 0 | 0 | 0 | 0 | 190 |
| LSM | 1 | 5 | 54 | 123 | 7 | 0 |
| Total calcium | 0 | 0 | 0 | 0 | 2 | 188 |
| Total cholesterol | 0 | 0 | 0 | 0 | 0 | 190 |
| Triglycerides | 0 | 0 | 0 | 0 | 0 | 190 |
| Uric acid | 0 | 0 | 0 | 0 | 0 | 190 |

Note: Values are numbers of patients. Each row totals 190 patients, and measurements were counted across the five scheduled visits.

**Supplementary Table S3C. Observations eligible for season-adjusted models at each visit**

| **Outcome** | **Baseline** | **Week 12** | **Week 24** | **Week 36** | **Week 48** |
| --- | --- | --- | --- | --- | --- |
| 25(OH)D | 190/190 | 189/190 | 189/190 | 189/190 | 189/190 |
| Total calcium | 190/190 | 188/189 | 189/190 | 188/189 | 189/190 |
| Glucose | 190/190 | 189/190 | 189/190 | 189/190 | 189/190 |
| Uric acid | 190/190 | 189/190 | 189/190 | 189/190 | 189/190 |
| Triglycerides | 190/190 | 189/190 | 189/190 | 189/190 | 189/190 |
| Total cholesterol | 190/190 | 189/190 | 189/190 | 189/190 | 189/190 |
| HDL-C | 190/190 | 189/190 | 189/190 | 189/190 | 189/190 |
| LDL-C | 190/190 | 189/190 | 189/190 | 189/190 | 189/190 |
| ALT | 190/190 | 189/190 | 189/190 | 189/190 | 189/190 |
| AST | 190/190 | 189/190 | 189/190 | 189/190 | 189/190 |
| CAP | 173/173 | 18/18 | 135/136 | 25/25 | 157/158 |
| LSM | 173/173 | 18/18 | 135/136 | 25/25 | 157/158 |
| HBsAg log10 | 190/190 | 189/190 | 189/190 | 189/190 | 189/190 |
| HBV DNA log10 | 190/190 | 38/38 | 132/133 | 29/29 | 148/149 |

Note: Values are model-eligible observations/observations with available outcome data. Model eligibility required a valid sampling date and complete model covariates. No missing values were imputed. Abbreviations are defined in Supplementary Table S3A.

**Supplementary Table S4. Observed laboratory and virological measurements by visit**

| **Outcome** | **Baseline** | **Week 12** | **Week 24** | **Week 36** | **Week 48** |
| --- | --- | --- | --- | --- | --- |
| 25(OH)D (nmol/L) | 68.5 [50.5, 86.4] | 62.5 [45.4, 80.3] | 55.0 [42.1, 71.0] | 54.1 [40.4, 69.1] | 57.6 [45.7, 76.8] |
| Total calcium (mmol/L) | 2.28 [2.25, 2.35] | 2.25 [2.20, 2.31] | 2.24 [2.18, 2.30] | 2.23 [2.16, 2.28] | 2.23 [2.18, 2.28] |
| Glucose (mmol/L) | 4.93 [4.67, 5.26] | 4.80 [4.63, 5.17] | 4.92 [4.68, 5.18] | 4.98 [4.72, 5.27] | 4.93 [4.69, 5.23] |
| Uric acid (µmol/L) | 339.5 [267.3, 386.5] | 325.5 [281.0, 383.0] | 328.0 [279.5, 385.3] | 321.0 [273.3, 369.3] | 326.5 [273.3, 377.8] |
| Triglycerides (mmol/L) | 1.12 [0.82, 1.76] | 1.51 [1.04, 2.11] | 1.48 [1.08, 2.04] | 1.45 [1.11, 2.05] | 1.44 [1.08, 1.93] |
| Total cholesterol (mmol/L) | 4.30 [3.70, 4.88] | 3.96 [3.43, 4.46] | 3.95 [3.32, 4.34] | 3.96 [3.42, 4.49] | 3.97 [3.39, 4.46] |
| HDL-C (mmol/L) | 1.31 [1.05, 1.66] | 1.15 [0.94, 1.35] | 1.14 [0.96, 1.38] | 1.16 [0.97, 1.38] | 1.12 [0.93, 1.39] |
| LDL-C (mmol/L) | 2.46 [2.05, 2.97] | 2.12 [1.74, 2.51] | 2.13 [1.73, 2.62] | 2.24 [1.79, 2.64] | 2.19 [1.74, 2.73] |
| ALT (U/L) | 27.0 [20.0, 41.8] | 60.0 [40.3, 98.8] | 52.5 [31.0, 78.0] | 40.0 [26.0, 65.8] | 36.0 [23.0, 59.0] |
| AST (U/L) | 25.5 [21.0, 33.0] | 52.0 [36.0, 78.8] | 46.0 [31.3, 66.0] | 39.0 [29.0, 56.0] | 36.0 [25.0, 50.8] |
| CAP (dB/m) | 224.0 [202.0, 260.0] | 241.5 [212.0, 273.5] | 234.0 [205.3, 266.0] | 208.0 [191.0, 251.0] | 234.5 [207.3, 261.0] |
| LSM (kPa) | 5.50 [4.80, 6.40] | 5.80 [4.73, 8.38] | 5.80 [4.90, 7.00] | 6.40 [5.20, 7.40] | 5.80 [5.00, 6.70] |
| HBsAg (log10 IU/mL) | 2.53 [1.66, 3.07] | 2.09 [0.52, 2.90] | 1.47 [−0.48, 2.61] | 1.25 [−0.89, 2.42] | 1.06 [−1.30, 2.45] |
| HBV DNA (log10 IU/mL) | 2.00 [2.00, 2.41] | 2.00 [2.00, 2.00] | 1.54 [1.30, 2.00] | 1.59 [1.30, 2.00] | 1.30 [1.30, 2.00] |

Note: Values are median [IQR] among available observations at each visit. Visit-specific sample sizes are reported in Supplementary Table S3A. Values below the HBsAg and HBV DNA assay limits were handled as described in the Methods before log10 transformation.

**Supplementary Table S5A. Visit-timing and random-effects sensitivity analyses**

| **Model** | **Patients** | **Observations** | **Adjusted 48-week change (95% CI), nmol/L** | **P value** |
| --- | --- | --- | --- | --- |
| Continuous time based on nominal visit week | 190 | 946 | −8.3 (−10.2 to −6.5) | <0.001 |
| Continuous time based on actual elapsed time | 190 | 946 | −7.8 (−9.4 to −6.1) | <0.001 |
| Categorical visit time; season based on scheduled dates | 190 | 946 | −7.6 (−9.6 to −5.7) | <0.001 |
| Categorical visit time; visits within ±8 weeks | 154 | 770 | −6.1 (−8.2 to −3.9) | <0.001 |
| Categorical visit time; visits within ±12 weeks | 178 | 890 | −7.5 (−9.5 to −5.5) | <0.001 |
| Categorical visit time; random-intercept model | 190 | 946 | −7.9 (−9.8 to −6.0) | <0.001 |
| Categorical visit time; random-intercept and linear random-slope model | 190 | 946 | −7.8 (−10.5 to −5.1) | <0.001 |

Note: Continuous-time models report cumulative 48-week trends, whereas categorical visit-time models report week-48 differences from baseline. All models were adjusted for seasonality, age, and sex. CI, confidence interval.

**Supplementary Table S5B. Comparison of linear and non-linear time specifications**

| **Comparison** | **AIC, linear model** | **AIC, alternative model** | **Likelihood-ratio statistic** | **df** | **P value** |
| --- | --- | --- | --- | --- | --- |
| Linear vs quadratic time | 7568.12 | 7543.00 | 27.12 | 1 | <0.001 |
| Linear vs categorical visit time | 7568.12 | 7545.83 | 28.29 | 3 | <0.001 |

Note: Models were fitted by maximum likelihood using 946 observations from 190 patients. Lower AIC indicates better relative fit. AIC, Akaike information criterion; df, degrees of freedom.

**Supplementary Table S5C. Actual elapsed time at each nominal visit**

| **Nominal visit week** | **25(OH)D observations** | **Valid sampling dates** | **Actual elapsed time from baseline, weeks** | | | | |
| --- | --- | --- | --- | --- | --- | --- | --- |
|  |  |  | **Minimum** | **Q1** | **Median** | **Q3** | **Maximum** |
| Baseline | 190 | 190 | 0.0 | 0.0 | 0.0 | 0.0 | 0.0 |
| Week 12 | 190 | 189 | 8.0 | 11.6 | 12.6 | 13.9 | 66.6 |
| Week 24 | 190 | 189 | 14.3 | 22.7 | 25.0 | 28.0 | 81.1 |
| Week 36 | 190 | 189 | 24.7 | 35.1 | 37.0 | 39.4 | 91.4 |
| Week 48 | 190 | 189 | 5.4 | 48.9 | 51.1 | 53.3 | 105.4 |

Note: Elapsed time was calculated in weeks from the recorded baseline sampling date. Four follow-up observations lacked a valid sampling date. Q1, first quartile; Q3, third quartile.

**Supplementary Table S5D. Leave-one-patient-out sensitivity analysis of the week-48 25(OH)D estimate**

| **Number of analyses** | **Minimum estimate, nmol/L** | **Maximum estimate, nmol/L** | **Largest P value** | **All 95% CI upper limits below 0** |
| --- | --- | --- | --- | --- |
| 190 | −8.2 | −7.6 | <0.001 | Yes |

Note: The adjusted categorical visit-time model was refitted 190 times, excluding one patient in each analysis. Estimates are week-48 differences from baseline. CI, confidence interval.

**Supplementary Table S5E. Cohort size and precision of principal estimates**

| **Quantity** | **Value** |
| --- | --- |
| Cohort size, n | 190 |
| Cohort basis | All retained participants from the consecutive cohort; no a priori sample-size calculation. |
| Adjusted categorical visit-time week-48 difference, nmol/L | −7.9 (95% CI, −9.8 to −6.0) |
| Spearman correlation between 48-week changes | 0.150 (bootstrap 95% CI, 0.009 to 0.290) |
| Approximate minimum detectable absolute correlation with 80% power | 0.202 |

Note: The bootstrap confidence interval was based on 2,000 patient-level resamples. The detectable correlation was calculated using Fisher’s z approximation with two-sided α = 0.05 and 80% power. CI, confidence interval.

**Supplementary Table S6A. Distribution of 25(OH)D concentrations using alternative cutoffs**

| **Visit** | **Patients, n** | **<50 nmol/L, n (%)** | **50 to <75 nmol/L, n (%)** | **≥75 nmol/L, n (%)** |
| --- | --- | --- | --- | --- |
| Baseline | 190 | 47 (24.7%) | 70 (36.8%) | 73 (38.4%) |
| Week 12 | 190 | 62 (32.6%) | 69 (36.3%) | 59 (31.1%) |
| Week 24 | 190 | 81 (42.6%) | 73 (38.4%) | 36 (18.9%) |
| Week 36 | 190 | 80 (42.1%) | 71 (37.4%) | 39 (20.5%) |
| Week 48 | 190 | 68 (35.8%) | 70 (36.8%) | 52 (27.4%) |

Note: Values are n (%).

**Supplementary Table S6B. Changes in proportions below 25(OH)D cutoffs from baseline to week 48**

| **25(OH)D cutoff (nmol/L)** | **Paired n** | **Below cutoff at baseline, n (%)** | **Below cutoff at week 48, n (%)** | **Newly below cutoff, n** | **Moved to ≥ cutoff, n** | **Exact McNemar P value** |
| --- | --- | --- | --- | --- | --- | --- |
| 47.7 | 190 | 40 (21.1%) | 66 (34.7%) | 37 | 11 | <0.001 |
| 50.0 | 190 | 47 (24.7%) | 68 (35.8%) | 33 | 12 | 0.002 |

Note: Newly below cutoff and moved to ≥ cutoff denote discordant baseline-to-week-48 transitions. P values were calculated using the exact McNemar test.

**Supplementary Table S6C. 25(OH)D changes by baseline tertile and regression-to-the-mean assessment**

| **Baseline tertile** | **N** | **Baseline, median [IQR]** | **Week 48, median [IQR]** | **Observed change, median [IQR]** | **Adjusted week-48 difference (95% CI)** |
| --- | --- | --- | --- | --- | --- |
| T1 (lowest) | 64 | 45.1 [38.7, 50.5] | 44.8 [35.3, 53.0] | −0.3 [−8.8, 13.4] | 2.0 (−1.1 to 5.2) |
| T2 | 63 | 68.6 [64.0, 72.6] | 57.2 [46.6, 69.7] | −9.9 [−21.2, 2.1] | −9.1 (−12.2 to −6.0) |
| T3 (highest) | 63 | 92.6 [86.7, 105.5] | 80.4 [64.2, 98.5] | −15.3 [−30.0, −3.5] | −16.7 (−19.9 to −13.6) |

Note: Values are expressed in nmol/L. The overall difference in observed changes across baseline tertiles was assessed using the Kruskal-Wallis test (P < 0.001). Adjusted differences were estimated using a visit-by-tertile mixed-effects model adjusted for seasonality, age, and sex. The Oldham correlation between within-patient change and the mean of baseline and week-48 values was ρ = −0.064 (P = 0.383). CI, confidence interval.

**Supplementary Table S6D. Baseline-to-week-48 transitions between 25(OH)D categories**

| **Baseline category** | **Week 48: <50 nmol/L** | **Week 48: 50 to <75 nmol/L** | **Week 48: ≥75 nmol/L** | **Total** |
| --- | --- | --- | --- | --- |
| <50 nmol/L | 35 | 11 | 1 | 47 |
| 50 to <75 nmol/L | 27 | 31 | 12 | 70 |
| ≥75 nmol/L | 6 | 28 | 39 | 73 |
| Total | 68 | 70 | 52 | 190 |

Note: Values are numbers of patients. Rows represent baseline categories and columns represent week-48 categories.

**Supplementary Table S7A. Visit-specific 25(OH)D differences under sequential covariate adjustment**

| **Visit** | **Unadjusted difference (95% CI)** | **Age- and sex-adjusted difference (95% CI)** | **Season-, age-, and sex-adjusted difference (95% CI)** |
| --- | --- | --- | --- |
| Week 12 | −6.2 (−8.1 to −4.2) | −6.2 (−8.1 to −4.2) | −5.2 (−7.3 to −3.1) |
| Week 24 | −11.4 (−13.4 to −9.5) | −11.4 (−13.4 to −9.5) | −8.6 (−10.9 to −6.3) |
| Week 36 | −12.6 (−14.6 to −10.7) | −12.7 (−14.6 to −10.7) | −10.4 (−12.5 to −8.2) |
| Week 48 | −7.8 (−9.7 to −5.8) | −7.8 (−9.7 to −5.8) | −7.9 (−9.8 to −6.0) |

Note: Values are differences from baseline in nmol/L (95% CI) estimated using categorical visit-time random-intercept models. All models included 190 patients and 946 observations. CI, confidence interval.

**Supplementary Table S7B. Seasonal model parameters and harmonic comparison**

| **Parameter** | **Estimate** | **P value** |
| --- | --- | --- |
| Annual sine coefficient | 1.2 | 0.030 |
| Annual cosine coefficient | −2.5 | <0.001 |
| Annual amplitude | 2.8 | - |
| Peak day of year | 157 | - |
| Approximate peak date | 5 Jun | - |
| Additional semi-annual harmonic, likelihood-ratio χ² (df = 2) | 3.72 | 0.156 |
| AIC: annual model / annual plus semi-annual model | 7545.83 / 7546.11 | - |

Note: Annual seasonality was represented by sin(2π × DOY/365.25) and cos(2π × DOY/365.25). The harmonic comparison used maximum-likelihood models with 946 observations from 190 patients. AIC, Akaike information criterion; DOY, day of year; df, degrees of freedom.

**Supplementary Table S7C. Calendar-month distribution of baseline sampling**

| **Month** | **Patients, n** | **%** |
| --- | --- | --- |
| Jan | 1 | 0.5 |
| Feb | 15 | 7.9 |
| Mar | 24 | 12.6 |
| Apr | 25 | 13.2 |
| May | 31 | 16.3 |
| Jun | 35 | 18.4 |
| Jul | 42 | 22.1 |
| Aug | 5 | 2.6 |
| Sep | 0 | 0.0 |
| Oct | 1 | 0.5 |
| Nov | 6 | 3.2 |
| Dec | 5 | 2.6 |

Note: Month was determined from the baseline sampling date (n = 190).

**Supplementary Table S8A. Residual diagnostics for outcome-specific mixed-effects models**

| **Marker** | **Model** | **Observations** | **Patients** | **Residual skewness** | **\|Normalized residual\| >3, %** | **Shapiro–Wilk P value** |
| --- | --- | --- | --- | --- | --- | --- |
| 25(OH)D | Categorical time | 946 | 190 | 0.08 | 0.74 | <0.001 |
| Total calcium | Continuous time | 944 | 190 | 2.57 | 0.85 | <0.001 |
| Glucose | Continuous time | 946 | 190 | 0.67 | 1.06 | <0.001 |
| Uric acid | Continuous time | 946 | 190 | 0.53 | 0.74 | <0.001 |
| Triglycerides | Continuous time | 946 | 190 | 5.64 | 1.80 | <0.001 |
| Total cholesterol | Continuous time | 946 | 190 | 0.29 | 0.63 | <0.001 |
| HDL-C | Continuous time | 946 | 190 | 1.55 | 0.63 | <0.001 |
| LDL-C | Continuous time | 946 | 190 | 0.29 | 0.32 | <0.001 |
| ALT | Continuous time | 946 | 190 | 4.00 | 1.37 | <0.001 |
| AST | Continuous time | 946 | 190 | 4.20 | 1.48 | <0.001 |
| CAP | Continuous time | 508 | 189 | 0.04 | 0.79 | <0.001 |
| LSM | Continuous time | 508 | 189 | 2.23 | 1.18 | <0.001 |
| HBsAg log10 | Continuous time | 946 | 190 | 0.19 | 0.42 | <0.001 |
| HBV DNA log10 | Continuous time | 537 | 190 | 3.22 | 2.05 | <0.001 |

Note: Results are based on normalized residuals from outcome-specific mixed-effects models adjusted for seasonality, age, and sex. The 25(OH)D model used categorical visit time, whereas secondary-outcome models used continuous time. Shapiro-Wilk P values are descriptive.

**Supplementary Table S8B. Log-transformed sensitivity models for skewed secondary outcomes**

| **Outcome** | **Patients** | **Observations** | **Week-48/baseline ratio (95% CI)** | **Percent change (95% CI), %** | **P value** |
| --- | --- | --- | --- | --- | --- |
| Triglycerides | 190 | 946 | 1.22 (1.15 to 1.30) | 21.9 (14.6 to 29.5) | <0.001 |
| ALT | 190 | 946 | 1.07 (0.96 to 1.18) | 6.8 (−3.6 to 18.3) | 0.207 |
| AST | 190 | 946 | 1.17 (1.07 to 1.27) | 16.6 (7.0 to 27.0) | <0.001 |
| LSM | 189 | 508 | 1.07 (1.03 to 1.11) | 7.0 (3.0 to 11.2) | <0.001 |

Note: Ratios and percentage changes represent model-based cumulative changes over 48 weeks from natural-log mixed-effects models adjusted for seasonality, age, and sex, with a patient-specific random intercept. P values are unadjusted. CI, confidence interval.

**Supplementary Table S9A. Spearman correlations between 25(OH)D and HBsAg**

| **Analysis** | **Patients, n** | **Spearman ρ** | **P value** | **FDR-adjusted P value** |
| --- | --- | --- | --- | --- |
| Baseline 25(OH)D and baseline HBsAg | 190 | −0.023 | 0.750 | 0.864 |
| Week-48 25(OH)D and week-48 HBsAg | 190 | 0.013 | 0.864 | 0.864 |
| Changes from baseline to week 12 | 190 | −0.026 | 0.727 | 0.864 |
| Changes from baseline to week 24 | 190 | 0.091 | 0.210 | 0.419 |
| Changes from baseline to week 36 | 190 | 0.154 | 0.034 | 0.117 |
| Changes from baseline to week 48 | 190 | 0.150 | 0.039 | 0.117 |

Note: HBsAg was log10-transformed after values below the assay limit were handled as described in the Methods. Changes were calculated as follow-up minus baseline. The six correlations were adjusted as one multiple-testing family using the Benjamini-Hochberg method. FDR, false discovery rate; HBsAg, hepatitis B surface antigen.

**Supplementary Table S9B. Sensitivity of the 48-week change correlation to HBsAg assay-limit handling**

| **Analysis** | **Method** | **Patients, n** | **Correlation coefficient** | **P value** |
| --- | --- | --- | --- | --- |
| All patients; below-limit HBsAg values set to 0.05 IU/mL | Spearman | 190 | 0.150 | 0.039 |
| All patients; below-limit HBsAg values set to 0.025 IU/mL | Spearman | 190 | 0.154 | 0.034 |
| Excluding patients with HBsAg <0.05 IU/mL at week 48 | Spearman | 140 | 0.101 | 0.237 |
| All patients; rank analysis with ties | Kendall | 190 | 0.100 | 0.040 |

Note: Correlations were calculated between baseline-to-week-48 changes in 25(OH)D and log10-transformed HBsAg. HBsAg results reported as <0.05 IU/mL were set to 0.05 IU/mL in the main analysis and 0.025 IU/mL in the half-limit sensitivity analysis; exact values of 0.05 IU/mL were unchanged. P values are unadjusted.

**Supplementary Table S9C. Concurrent within- and between-patient associations between 25(OH)D and HBsAg**

| **Model** | **25(OH)D component** | **Patients, n** | **Observations, n** | **β (95% CI)** | **P value** |
| --- | --- | --- | --- | --- | --- |
| Reporting-limit substitution; random intercept | Within-patient | 190 | 946 | 0.068 (0.020 to 0.116) | 0.006 |
| Reporting-limit substitution; random intercept | Between-patient | 190 | 946 | 0.018 (−0.088 to 0.123) | 0.746 |
| Half-limit substitution; random intercept | Within-patient | 190 | 946 | 0.073 (0.022 to 0.124) | 0.005 |
| Half-limit substitution; random intercept | Between-patient | 190 | 946 | 0.015 (−0.095 to 0.126) | 0.788 |
| Left-censored Gaussian model, cluster-robust SE | Within-patient | 190 | 946 | 0.075 (0.003 to 0.147) | 0.041 |
| Left-censored Gaussian model, cluster-robust SE | Between-patient | 190 | 946 | 0.016 (−0.120 to 0.152) | 0.821 |

Note: β represents the difference in log10 HBsAg associated with a 10-nmol/L higher 25(OH)D concentration. All models adjusted for categorical visit time, seasonality, age, and sex. Substitution models included a patient-specific random intercept; the left-censored model used patient-cluster robust standard errors. HBsAg values reported as <0.05 IU/mL were treated as left-censored, whereas exact values of 0.05 IU/mL were uncensored. P values are unadjusted. CI, confidence interval; SE, standard error.

**Supplementary Table S9D. HBsAg loss and longitudinal patterns through week 48**

| **Outcome** | **n/N (%)** |
| --- | --- |
| HBsAg loss at baseline | 0/190 (0.0%) |
| HBsAg loss at week 48 | 50/190 (26.3%) |
| Week-48 loss also present at week 36 | 41/50 (82.0%) |
| Week-48 loss documented at two consecutive visits | 41/50 (82.0%) |
| First observed loss at week 48 | 7/50 (14.0%) |
| HBsAg reappearance after an earlier loss | 3/190 (1.6%) |

Note: HBsAg loss was defined as HBsAg <0.05 IU/mL. HBsAg reappearance was defined as a subsequent value ≥0.05 IU/mL after an earlier loss. HBsAg, hepatitis B surface antigen.

**Supplementary Table S9E. HBsAg results below and at the assay limit by visit**

| **Visit** | **Available, n** | **HBsAg <0.05, n (%)** | **Exact HBsAg 0.05, n (%)** | **HBsAg ≤0.05, n (%)** |
| --- | --- | --- | --- | --- |
| Baseline | 190 | 0 (0.0%) | 2 (1.1%) | 2 (1.1%) |
| Week 12 | 190 | 13 (6.8%) | 0 (0.0%) | 13 (6.8%) |
| Week 24 | 190 | 34 (17.9%) | 0 (0.0%) | 34 (17.9%) |
| Week 36 | 190 | 41 (21.6%) | 0 (0.0%) | 41 (21.6%) |
| Week 48 | 190 | 50 (26.3%) | 0 (0.0%) | 50 (26.3%) |

Note: HBsAg results reported as <0.05 IU/mL were classified as below the assay limit; exact values of 0.05 IU/mL were not. The main and half-limit substitutions were 0.05 and 0.025 IU/mL, corresponding to −1.30 and −1.60 log10 IU/mL, respectively. HBsAg, hepatitis B surface antigen.

**Supplementary Table S10. Associations between baseline 25(OH)D and week-48 HBsAg outcomes**

| **Outcome** | **Baseline 25(OH)D exposure** | **Participants, n** | **Events, n** | **Adjusted OR (95% CI)** | **P value** |
| --- | --- | --- | --- | --- | --- |
| HBsAg decline ≥1 log10 IU/mL by week 48 | <47.7 versus ≥47.7 nmol/L | 190 | 104 | 0.94 (0.41 to 2.18) | 0.893 |
| HBsAg decline ≥1 log10 IU/mL by week 48 | Per 10-nmol/L increase | 190 | 104 | 0.99 (0.86 to 1.15) | 0.938 |
| HBsAg loss at week 48 | <47.7 versus ≥47.7 nmol/L | 190 | 50 | 2.28 (0.68 to 7.59) | 0.180 |
| HBsAg loss at week 48 | Per 10-nmol/L increase | 190 | 50 | 1.01 (0.84 to 1.23) | 0.884 |

Note: Each row represents a separate logistic regression model adjusted for baseline HBsAg, age, sex, and baseline seasonal sine and cosine terms. HBsAg loss was defined as HBsAg <0.05 IU/mL. P values are unadjusted. CI, confidence interval; OR, odds ratio.
